# Genomic characterization, high-density mapping and anchoring of DArT markers to the reference genome of *Eucalyptus*

**DOI:** 10.1186/1753-6561-5-S7-P35

**Published:** 2011-09-13

**Authors:** César Petroli, Carolina Sansaloni, Jason Carling, Eva M C Mamani, Dorothy A Steane, Alexander A Myburg, René E Vaillancourt, Andrzej Kilian, Georgios J Pappas, Orzenil Bonfim da Silva, Dario Grattapaglia

**Affiliations:** 1 EMBRAPA Genetic Resources and Biotechnology - EPqB Final W5 Norte 70770-917 Brazilia DF and Dep. Cell Biology, Universidade de Brazilia – UnB, Brazil; 2 Diversity Arrays Technology Pty Ltd. 1 Wilf Crane Crescent, Yarralumla, ACT 2600, Australia; 3 School of Plant Science and CRC for Forestry, University of Tasmania, Private Bag 55. Hobart, Tasmania 7001. Australia; 4 Department of Genetics, Forestry and Agricultural Biotechnology Institute (FABI). University of Pretoria. Pretoria. 0002. South Africa; 5 EMBRAPA Genetic Resources and Biotechnology - EPqB Final W5 Norte 70770-917 Brazilia DF, Brazil

## Background

Genetic linkage maps have been essential tools to examine the inheritance of qualitative and quantitative traits, to carry out comparative mapping and to provide markers for molecular breeding applications. Linkage maps for species of Eucalyptus have been reported for several pedigrees using different molecular marker technologies [1]. However improved marker density, throughput and transferability across species are necessary to increase resolution of current maps for a variety of genomic applications. We report the development of a high density linkage map for Eucalyptus based on microsatellites and DArT (Diversity Arrays Technology) markers generated by a standardized genotyping microarray [2]. DNA probes that constitute the DArT microarray were sequenced and positioned on the reference Eucalyptus genome providing information about their sequence content, their distribution relative to annotated genes as well as the relationship between physical and recombination distance in the Eucalyptus genome.

## Methods

Map construction was carried out using an F1 progeny of 177 individuals derived from a cross between *E.grandis* and *E.urophylla*. Genomic representations of both parents and their F1 offspring were produced with the same complexity reduction method used to prepare the library (PstI/TaqI) generating the ‘targets’ for hybridizing to the arrays. Microarray imaging, data extraction polymorphism detection and marker scoring were carried out using *DArTSoft* v.7.44 (http://www.diversityarrays.com/). Polymorphic markers were filtered according to reproducibility, quality parameter (Q) and marker call rate as described earlier [2]. An integrated linkage map was constructed with JoinMap v3.0 [3]using a framework map of previously mapped fully informative anchor microsatellites. All 7,680 DNA probes that constitute the current DArT genotyping microarray were Sanger sequenced. Redundancy analysis was carried out at the sequence level using a 50bp minimum overlap, 98% identity, and allowing for 10% mismatch and gap size of one bp. These DArT probes were mapped on the annotated Eucalyptus reference genome as available in Phytozome. Distribution of DArT probes and mapped DArT markers relative to the predicted gene models was carried out by dividing the genome in 500 kbp bins corresponding approximately to a 1 cM recombination fraction.

## Results

A segregation ratio filtering (both for 1:1 and 3:1 markers) together with a relaxed call rate threshold > 50% were initially applied to include the largest number of potentially mappable markers. A total of 4,271 DArT markers segregated 1:1 and 1,572 segregated 3:1. The complete dataset with 6,065 markers (5,843 DArT and 222 microsatellites) was submitted to a linkage analysis resulting in eleven groups at LOD ≥ 15 with a total of 2,484 markers. A subset of 1,032 markers positioned at high likelihood for ordering (864 DArTs and 168 microsatellites) provided a framework map with 1,176 cM and an average recombination distance between consecutive markers of 1.15 cM. When all the 2,484 mapped markers were fitted, the total recombining genome length increased to 1,303 cM with a resolution of 0.6 cM. A redundancy analysis of the 6,918 DArT probes for which sequences could be obtained resulted in 4,583 unique sequences (66%). The estimated redundancy of 34% represents a useful resource by providing alternative probes for detecting polymorphism in the same genomic region in different individuals. A total of 6,480 probes (93.7%) were confidently mapped onto the reference genome; 4,189 of them (65%) mapped to single positions while the remaining 2,291 probes mapped to a second position with a relatively high suboptimal alignment score and were therefore considered mapped at lower confidence. About 50% of these low confidence mapping (1,026) actually mapped to a sequence position containing a repeat element which might explain the mapping result. A total of 438 DArT probes could not be mapped to the current assembly. These DArT probes might further improve the current genome version by including some yet unassembled genome contigs. A total of 1,987 linkage mapped DArT markers for which sequence was available were positioned relative to the 41,208 gene models in the current genome, distributed in 500 kbp bins providing an average of 1.6 ± 2.4 DArT markers for the 34 ± 15 gene models present in each genome bin. Interestingly a total of 4,663 DArT probes (67%) mapped at less than ten basepairs from the nearest gene model and only 76 probes mapped at more than 10kbp. The largest distance from a DArT probe to the next gene was only 156 kbp and a modest although highly significant coincidence across the whole genome was seen between the number of DArT markers and the number of gene models (Spearman rank correlation R = 0.33 p<0.00000).

## Conclusions

The linkage map presented in this work was used to aid the ordering of contigs during the assembly of the *Eucalyptus grandis* genome currently available through the JGI Phytozome website. This map, together with other parallel mapping efforts that used this same genotyping platform have provided between 2,000 and 3,000 segregating markers irrespective of species of Eucalyptus. The mapping density achieved with the Eucalyptus DArT microarray provides unprecedented opportunities for comparative mapping across pedigrees, high resolution QTL analysis and molecular breeding applications across species of the genus. We have shown that not only the DArT marker sequences are highly enriched for genes, but that their distribution relative to the predicted gene models provides an extremely efficient tool to specifically tag genes at distances within the extent of LD in typical breeding populations. The use of this standardized high-throughput genotyping platform will therefore be instrumental to implement genome-wide selection strategies and positional cloning efforts in multiple Eucalyptus species.
